# Analysis of acceptance of the smoking ban in Greece

**DOI:** 10.1186/1617-9625-12-S1-A21

**Published:** 2014-06-06

**Authors:** Efthimios Zervas, Nikolaos Papantonis, Eleni Ischaki, Eleni Litsiou, Paraskevi Katsaounou

**Affiliations:** 1 School of Science and Technology, Hellenic Open University, Greece; 2 Pulmonary department-ICU, Εvaggelismos Hospital, Athens, 10676, Greece

## Background

Exposure to secondhand smoke, according to the World Health Organization is responsible for 603,000 deaths in 2004. The ban on smoking in public places in Greece was legislated but not implemented, as the adequate and effective measures were not taken. This study aims to determine the degree of acceptance of the last anti-smoking law and the parameters associated with the acceptance or not of the smoking ban.

## Materials and methods

The survey was based on 540 valid questionnaires collected between February and April 2011 in Attica. The statistical analysis is initially focused on the mean value and the distribution of responses concerning the smoking habits and the views about smoking restriction in enclosed public places. Then, the responses between smokers and non-smokers are separated and the correlation of responses with the personal data of respondents and between them is performed.

## Results

42% of respondents are smokers and 20% of them have a high degree of dependence. Only 9 % of respondents consider that the measures are sufficient for the protection of non-smokers, while 65 % agreed to a total smoking ban in enclosed spaces. 50 % believe that the law will not be implemented. 66 % believe that the ban would not affect the number of cigarettes smoked and do not consider that this ban leads to social discrimination against smokers. 75 % believe that the smoking ban will affect their mode of entertainment. In all questions the ban acceptability of smokers is reduced compared to non-smokers and also depending on the degree of addiction to smoking. The ban acceptance increases with the educational level.

## Conclusions

The Greek public, including smokers, is mature to accept the ban on smoking in public places. The respondents wait for the political will to implement this ban and the higher information of the public about the effects of passive smoking.

